# Coaches’ Corrective Feedback, Psychological Needs, and Subjective Vitality in Mexican Soccer Players

**DOI:** 10.3389/fpsyg.2020.631586

**Published:** 2021-02-04

**Authors:** José Tristán, Rosa María Ríos-Escobedo, Jeanette M. López-Walle, Jorge Zamarripa, Miguel A. Narváez, Octavio Alvarez

**Affiliations:** ^1^Faculty of Sports Organization, Autonomous University of Nuevo León, San Nicolás de los Garza, México; ^2^Department of Kinesiology, Western Illinois University, Macomb, IL, United States; ^3^Faculty of Psychology, University of Valencia, Valencia, Spain

**Keywords:** corrective feedback, basic psychological needs, subjective vitality, serial mediation model, legitimacy of feedback

## Abstract

In the sport context, an essential aspect of an athlete’s development and performance happens during the interaction with the coach while receiving information on the aspects of performance that need to be modified (corrective feedback). Grounded in the Self-Determination Theory and particularly on the basic psychological needs theory, a structural equation model (SEM) was tested with the following sequence: perception of the amount of corrective feedback generated by the coach, perceived legitimacy of corrective feedback, satisfaction of basic psychological needs, and vitality in soccer players. Additionally, simple mediation and serial (double) mediation models were also tested. Participants were 377 Mexican soccer players (*M_aged_* = 16.46, *SD* = 1.08), who completed the instruments that evaluated the study variables. SEM results reported positive and significant variables’ interrelations in the sequence. The analysis of serial mediation model showed that the perceived legitimacy of feedback and the satisfaction of basic psychological needs fully mediated the relationship between the perception of the amount of corrective feedback generated by the coach and the perception of the subjective vitality of Mexican soccer players. Results suggest that coaches have to ensure that athletes accept the corrective feedback provided and meet their basic psychological needs. Based on SDT tenets, this research highlights the importance for coaches to be aware of the athlete’s perceptions when they are providing corrective feedback and their implications for athlete’s technical development and well-being. It is suggested to incorporate those aspects to training programs for coaches.

## Introduction

Coaches play a fundamental role in athletes’ social environment by facilitating their learning, by monitoring their progress, and by providing adequate feedback that should lead them to improve their performance, cognitive learning (Nicaise et al., 2007), value the experience, and their behavior in sport (Carpentier and Mageau, 2016).

Clear communication of directions and expectations, as well as conveying that error is a part of learning (Smith et al., 2015), allowing athletes’ to enjoy what they do and enhancing the quality of their participation in sport. Corrective feedback (CF) is provided through statements that convey information on how to improve, after making mistakes or a bad performance (Mouratidis et al., 2010). CF focuses more on the qualitative aspects of the performance or processes in which the athlete has failed, in order to do it right in the future.

It is important for a coach to know that CF in sport is vital and difficult to provide. Nevertheless, corrections has been identified in previous literature as a good and necessary coach’ behavior (e.g., Álvarez et al., 2010). What literature underlined was not only *what*, but also *how* CF is provided and perceived by actors (i.e., athletes and coaches), the form in which coaches communicates CF has varied effects depending on how is perceived by athletes (Mouratidis et al., 2010). Such perception may cause different motivational states and diverse results on athletes’ behavior in sport (Hein and Koka, 2007; Mouratidis et al., 2010). Hence, the need in sport for tools that help coaches to provide such feedback maximizing its potential positive effects and minimizing the negative ones (Carpentier and Mageau, 2013).

Research has shown that perceived legitimacy, or the degree of acceptance of CF, is critical to CF’s effectiveness within a learning environment (Mouratidis et al., 2010; Tristán et al., 2017, 2018). However, studies on negative feedback in sport have yet to focus on the relevance of athletes’ perceived legitimacy and its association with basic psychological needs (BPNs). Furthermore, CF may have negative effects on students’ self-esteem, self-efficacy, and motivation to learn (Kim, 2004; Hattie and Timperley, 2007; Shute, 2008; Voerman et al., 2012).

The Self-Determination Theory (SDT) encompasses the Basic Psychological Needs Mini-Theory (BPNT; Deci and Ryan, 2002; Ryan and Deci, 2017), which posits that human beings possess universal needs to perceive competence, autonomy, and relatedness. The need for competence is defined as the desire to interact efficiently with the environment; the need for autonomy defined as the desire to self-organize experience and behavior and to have activity be concordant with one’s integrated sense of self; and the need for relatedness defined as the desire to be connected to others – to love and care and to be loved and care for. The BPNT proposes that satisfying the BPN improves the psychological well-being (Deci and Ryan, 2000; Ryan and Deci, 2017). Moreover, BPNT postulates that achieving well-being or ill-being is a function of the social environment and its potential to satisfy BPNs.

From the eudaimonic perspective, psychological well-being describes a person who is active, ambitious, and interested in developing his/her skills and potential. Hence, well-being equates to optimal growth and development (Ryan and Deci, 2001). Subjective vitality, defined as “a positive feeling of aliveness and energy” (Ryan and Frederick, 1997), is a variable used frequently as an indicator of eudaimonic well-being. The SDT states that, in addition to being predictors of well-being and influenced by social context, BPNs are mediators of social factors (e.g., the environment created by the coach) and psychological well-being (e.g., subjective vitality and life satisfaction).

Research within SDT has seldom studied CF, players’ legitimate perception (perceived legitimacy) and their relation to the satisfaction of BPNs in the context of sport, even though when, according to SDT, BPNs mediate athletes’ motivation and psychological well-being. Thus, it is important to understand how a coach should communicate CF to players, so that it is perceived by them as legitimate and can satisfy their BPNs. This is more relevant in sport when the aim is for players’ perception of CF, regardless of whether it conveys messages of low competence, is to not generate feelings of incompetence nor undermine the intrinsic motivation of players.

There is a body of knowledge that providing evidence of a positive relationship between CF supporting autonomy and the satisfaction of BPNs. Among these are the SDT (Deci and Ryan, 1985, 1991), BPNT principles (Deci and Ryan, 2002; Ryan and Deci, 2017), and research in the fields of physical education (Vergara-Torres et al., 2020) and sports (Mouratidis et al., 2010; Carpentier and Mageau, 2013, 2016; Tristán et al., 2017, 2018). Furthermore, previous research has shown a positive relationship of autonomy-supporting CF with autonomous motivation (Mouratidis et al., 2010; Carpentier and Mageau, 2016) and external regulation. Moreover, future intention to continue exercising, well-being (Mouratidis et al., 2010), and self-esteem (Carpentier and Mageau, 2016) have shown a positive relationship with autonomous motivation and external regulation. In contrast, negative relationships has been reported between ill-being (Mouratidis et al., 2010), autonomous motivation, and external regulation. Additionally, Mouratidis et al. (2010) reported that these relationships were partially mediated by the perceived legitimacy of CF (the degree of acceptance to CF). In the context of the physical education, when students perceive CF as legitimate, they perceive it as a satisfaction of their BPNs and report high levels of subjective vitality (Vergara-Torres et al., 2020).

The contexts of sport and physical education share common features, but are also distinct and each has unique attributes (Tristán et al., 2016). However, understanding the phenomena in physical education may provide an insight into sport, thus making it relevant (Vergara-Torres et al., 2020). Regardless of the fact that the hypothesized model has been tested in the context of physical education (Vergara-Torres et al., 2020), there is a gap in the literature about the effects that CF might have on satisfaction of BPNs and on the perceptions of vitality within the sport.

**Figure 1:**
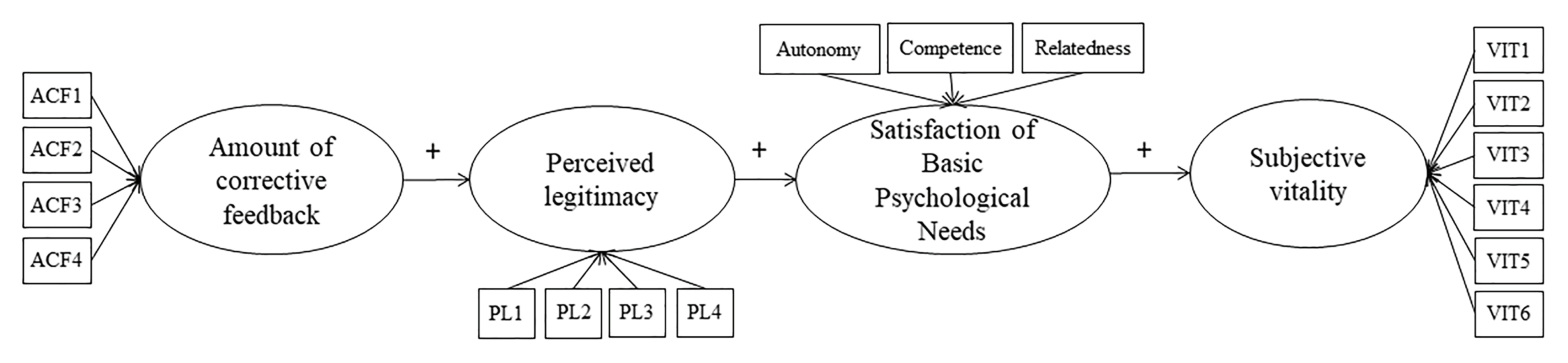
Hypothesized model of the relationship between social factor, personal mediating factors, and well-being of soccer players.

In sum, athletes value positively their coaches when they provide corrections that are clue to improve their sport level (e.g., Álvarez et al., 2010). In general and specifically in learning levels of competition (including junior level), coaches practice tends to reproduce a “traditional” approach to coaching behavior (based on instruction; e.g., Cushion et al., 2012) as probably they learned when they were athletes. Therefore, it would be interesting to explore if those coaches behaviors (i.e., CF) and well-being of athletes are mediated by personal variables (i.e., perceived legitimacy of feedback and satisfaction of BPN) in a sample in of Mexican adolescent athletes. To this end, and using the hierarchic model of intrinsic and extrinsic motivation proposed by Vallerand (1997) as a framework in combination with the review of literature, the purposes of this research were to study the relationships between social factor (perception of the amount of CF provided by the coach), personal factors (perceived legitimacy of feedback and satisfaction of BPNs – autonomy, competence, and relatedness), and well-being (subjective vitality) of young soccer players. Additionally, we analyzed the mediating role of perceived legitimacy of feedback and the satisfaction of BPNs on social factor and well-being (see Figure 1).

## Materials and Methods

### Participants

The sample included 377 youth male soccer players (*M*_*age*_ = 16.46; *SD* = 1.08), who were the part of representative teams from college preparatory schools or semi-professional teams. Participants were a convenience sample reflecting 27.4% of the total of representative college preparatory schools players. The inclusion criteria considered the participants who trained more than 3 days a week (*M* = 3.7; *SD* = 1.2), in training sessions longer than 2 h (*M* = 2.2; *SD* = 1.1) and had been competing under the guidance of the same coach for an average of 1.2 years (*SD* = 0.4). The sampling error was 4%.

### Instruments

The CF Scale (Mouratidis et al., 2010) in the contextualized Mexican version (Tristán et al., 2012) was used to measure the perceptions of CF received by the players and their perceived legitimacy. This scale is composed of four subscales with four items each (16 items in total), which include CF scale, perceived legitimacy, opportunity to learn, and perception illegitimate. For the purpose of this study, only the CF and perceived legitimacy subscales were used. All items were answered using a Likert response scale in a range of 1 (*completely disagree*) to 5 (*completely agree*). In every item, participants answered the question “To what degree is this true regarding your coach?” An example of an item in the CF subscale is “my coach points out my defects.” In the perceived legitimacy subscale, an example of an item is “I think that if my coach points out my defects, it is because he/she has a reason for it.” Previous studies have shown internal consistency in both scales (Mouratidis et al., 2010; Tristán et al., 2012).

Three instruments were used to evaluate BPN. Satisfaction of the need for Competence was evaluated with the Perceived Competence subscale from the Intrinsic Motivation Inventory (McAuley et al., 1989) in its Spanish version (Balaguer et al., 2008) and applied within the Mexican context (López-Walle et al., 2012). The inventory is composed of five items that evaluate perceived competency in the sport context. Responses are collected in a 7-point Likert-scale, ranging from 1 “in total disagreement” to 7 “in total agreement.” Previous research has confirmed the reliability of this scale in various languages such as Swedish (Patall et al., 2014) and French (Mascret et al., 2014). Items in the scale of perceived competency read as the following example, “I think that I’m really good at my sport.” Satisfaction of the need for Autonomy was evaluated using, the Perceived Autonomy in Sport Scale (Reinboth and Duda, 2006) in its validated Spanish version (Balaguer et al., 2008) and applied within the Mexican context (López-Walle et al., 2012). The scale has 10 items that start with the phrase “In my sport…” A complete item will read as “I can give my opinion.” Responses are collected in a 7-point Likert-scale ranging from 1 ‘not true” to 7 “true.” Finally, satisfaction of the need for Relatedness was evaluated using the Perceived Relatedness Scale (Perceived Relatedness Scale; Richer and Vallerand, 1998) in its Spanish version (Balaguer et al., 2008) and applied within the Mexican context (López-Walle et al., 2012). The scale has five items that start as follows “When I participate in my sport, I feel….” A complete item will read as “supported.” The Likert-scale ranges from 1 “totally disagree” to 5 “totally agree.” To measure satisfaction of BPNs, the average was calculated from the 20 items within the three scales (5 competence, 10 autonomy, and 5 relatedness), since the three variables share a significant percentage of the variance. This procedure has been proposed by various researchers (e.g., Ntoumanis, 2005; Standage et al., 2005; Álvarez et al., 2009; López-Walle et al., 2009). Subjective vitality was evaluated using the Subjective Vitality Scale (Ryan and Frederick, 1997) in its Spanish version (Castillo et al., 2017) and applied within the Mexican context (López-Walle et al., 2012). The scale has six items and measures the subjective feelings of energy and liveliness. Participants specify the degree to which a series of affirmations are true for them. An example of item is “I feel alive and vital.” The answers are collected in a 7-point Likert-scale ranging from 1 “not true” to 7 “totally true.” Previous research has confirmed the validity and reliability of this instrument (Ryan and Frederick, 1997; López-Walle et al., 2012; Castillo et al., 2017).

### Procedures

The research project followed research ethics guidelines by the American Psychology Association (APA). Permission to contact participants was obtained from the Department of Athletics as well as from every team coordinator and manager of participating high schools. All team coaches were informed in a personal interview about the research goals and the relevance of participating in the research project. Data were collected in each of the facilities, where teams practiced, in rooms made available by high schools for this purpose. Prior to administering questionnaires, participants received instructions about the research project and were informed that help was available if needed; at least one researcher was present in the classroom. Participation was voluntary, and the data collection was confidential and anonymous; coaches and team personnel were restricted from being present at the time of data collection.

### Data Analysis

Descriptive statistics were calculated for all the variables (means, standard deviations, and correlations) as well as scales’ internal consistency *via* Cronbach’s alpha coefficient. The structural equations model was tested with the study’s goal sequence, *via* AMOS 21.0 software, mediating models were estimated with the regression analysis macro PROCESS (Hayes, 2013) for SPSS.

The structural equations model adjustment was tested, considering various indices, which included chi-square (*χ*^2^) divided by degrees of freedom (Wheaton et al., 1977), the incremental fit index (Incremental Fit Index, IFI; Bentler and Bonett, 1980), the comparative fit index (Comparative Fit Index, CFI; Bentler, 1990), and the root-mean-square error of approximation (Root Mean Square Error of Approximation, RMSEA; Steiger and Lind, 1980). According to Carmines and McIver (1981), a quotient (*χ*^2^/df) less than six is a good model fit. CFI and NFI values over 0.90 indicate acceptable values (Hu and Bentler, 1995). For RMSEA, according to Browne and Cudeck (1993), a value of 0.08 or less indicates a reasonable error, and values greater than 1 are not admissible.

Model 6 of the macro PROCESS was used to analyze the simple and serial mediations (double) of perceived legitimacy and satisfaction of BPNs on the amount of CF and subjective vitality. To test indirect effects, bootstrap confidence intervals corrected for bias were used with 5,000 replications and 95% CI. This method requires the calculation of the product of regression coefficients that estimate the indirect effect and to obtain a CI for such effect. The mediation effect is confirmed when the confidence interval does not include zero (Hayes, 2013).

## Results

Internal consistency of instrumentation was satisfactory, with values between 0.70 and 0.94 (see Table 1). Item 4 was removed from the legitimate feedback perception variable due to its low discrimination index (< 0.30), which in turn increased the reliability of the instrument. Similarly, all study variables had a significant positive correlation between them (*p* < 0.01; see Table 1).

**Table 1:** Descriptive statistics, internal consistency and correlations between study variables.

Variables	Range	*M*	*SD*	1	2	3	4	5	6	7
1. Amount of corrective FB	1–5	3.46	0.80	0.70						
2. Perceived legitimacy of FB	1–5	3.80	0.76	0.59^**^	.70^a^					
3. SBPN Autonomy	1–7	5.29	1.07	0.21^**^	0.31^**^	0.93				
4. SBPN Competence	1–7	5.76	1.06	0.21^**^	0.32^**^	0.37^**^	0.88			
5. SBPN Relatedness	1–5	4.27	0.81	0.20^**^	0.26^**^	0.45^**^	0.40^**^	0.94		
6. SBPN	1–5	5.11	0.77	0.27^**^	0.39^**^	0.79^**^	0.77^**^	0.75^**^	-	
7. Subjective vitality	1–7	5.99	0.93	0.15^**^	0.28^**^	0.48^**^	0.36^**^	0.48^**^	0.56^**^	0.88

FB, feedback; SBPN, satisfaction of basic psychological needs. The correlation diagonal shows the Cronbach’s alpha reliability coefficient.

a Item 4 was not included.

** *p* < 0.001.

**Figure 2:**
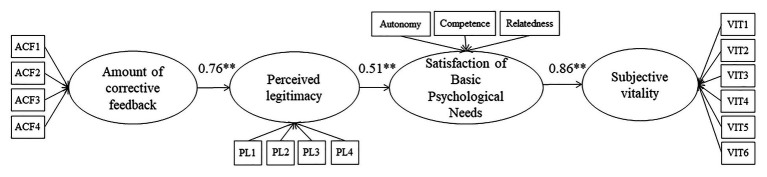
Standardized solution of the hypothesized structural model. ^**^p < 0.001.

The standardized solution of the hypothesized model indicates that the perception of the amount of CF provided by the coach has as positive relationship with the perceived legitimacy of feedback (*β* = 0.76, *p* < 0.01), which in turn correlates positively with the satisfaction of BPNs (*β* = 0.51, *p* < 0.01). Satisfaction of psychological needs positively correlates with participants’ subjective vitality (*β* = 0.86, *p* < 0.01). The model presents adequate goodness of fit indices *χ*^2^ = 385.8, gl = 116, *χ*^2^/gl = 3.32, *p* > 0.01, CFI = 0.89, IFI = 0.89, RMSEA = 0.08 (see Figure 2).

Analyses of mediating models show that the first simple mediation analysis was not significant, indicating that the perceived legitimacy of feedback dose not mediate the relationship between the perception of amount of CF and subjective vitality. The second serial mediation (double) analysis was significant, indicating that perceived legitimacy of feedback and the satisfaction of basic needs are mediators for amount of CF and subjective vitality. Lastly, the third analysis of simple mediation was not significant, indicating that the basic needs satisfaction does not mediate the relationship between amount of CF and subjective vitality.

In the serial mediation (double), the direct effects between perception of amount of CF and subjective vitality are canceled by the mediation of perceived legitimacy and the satisfaction of BPNs, confirming a total mediation of these variables (see Table 2).

**Table 2:** Complex mediation model: perceived legitimacy of feedback and basic psychological needs satisfaction as mediator between amount of corrective feedback and subjective vitality.

DV/*Predictor*	B	SE	LL CI 95%	UL CI 95%	*R*^2^
Perceived legitimacy of FB					0.34^**^
*Amount of corrective FB*	0.55^**^	0.04	0.47	0.63	
BPN satisfaction					0.14^**^
*Perceived legitimacy of FB*	0.34^**^	0.06	0.22	0.46	
*Amount corrective FB*	0.06	0.06	−0.05	0.17	
Subjective vitality					0.32^**^
*Perceived legitimacy of FB*	0.12	0.07	−0.02	0.25	
*BPN satisfaction*	0.65^**^	0.06	0.54	0.77	
*Amount corrective FB*	−0.04	0.06	−0.16	0.08	
Direct effect	B	SE	LL CI 95%	UL CI 95%	*R*^2^
Subjective vitality					0.02^*^
*Amount of corrective FB*	0.19^*^	0.06	0.07	0.30	
Indirect effect (mediator)	B	SE	LL CI Bootstrap 95%	UL CI Bootstrap 95%	
(Perceived legitimacy of FB)	0.06	0.04	−0.00	0.14	
(Perceived legitimacy of FB and BPN satisfaction)	0.12	0.03	0.07	0.20	
(satisfaction BPN)	0.04	0.04	−0.03	0.12	

FB, feedback; BPN, basic psychological needs; DV, dependent variable; B, non-standardized regression coefficient; SE, standard error; LL, lower limit; UL, upper limit; CI, confidence interval.

** *p* < 0.001;

* *p* < 0.01.

## Discussion

This study tested the BPNT (Deci and Ryan, 2002; Ryan and Deci, 2017), derived from the Self Determination Theory (Deci and Ryan, 1985; Ryan and Deci, 2017) in the sport context, specifically in Mexican youth soccer. We analyzed the relationships of perception of amount of CF provided by the coach, perceived legitimacy of CF, satisfaction of BPNs, and subjective vitality of youth soccer players. Additionally, we studied the mediating roles of the perceived legitimacy of CF and the satisfaction of BPNs, individually and as a group (serial) between CF and vitality. Results support the proposed sequence of BPNT in the sport context of soccer at the high school level.

Results confirm the first expected association between amount of CF and the perceived legitimacy of CF and their effects on the satisfaction of BPNs of soccer players and are in agreement with previous studies (Tristán et al., 2017; Vergara-Torres et al., 2020). Coaches need to know how to provide CF with reasonable, meaningful, credible, and specific justification. They need to be clear and precise and give players enough time to improve. These are effective strategies that prevent athletes from being discouraged by the corrective information provided (Mouratidis et al., 2010; Tristán et al., 2017, 2018) and will lead to the acceptance of the provided feedback and to its perception as legitimate. All this will be conducive for students and athletes to feel effective in their interactions in the social environment and to demonstrate confidence in achieving the desired results. By perceiving the CF received as legitimate, athletes will feel more autonomous, competent, and related to both their teammates and their coach. They will not feel discouraged or forced to continue in training or competitions by the corrective information provided (Amorose and Weiss, 1998; Mouratidis et al., 2010; Tristán et al., 2018).

In relation to previous research in the sport context (e.g., Reinboth and Duda, 2006; López-Walle et al., 2012), this study confirms the positive association between satisfaction of basic psychologic needs and subjective vitality. This means that athletes feel vital and full of positive energy, mainly due to the satisfaction of their BPNs. Therefore, it is suggested that coaches might pay attention to satisfy the needs of competence, autonomy, and relatedness of youth soccer players, in order to enhance their positive experiences during practices and competitions (Balaguer et al., 2012).

In regards to mediation results, the sequence of perceived legitimacy of feedback and the satisfaction of BPNs as total mediators of the relationship between the perception of the amount of CF provided by the coach and subjective vitality of athletes stands out.

In youth sports, most of the coaches’ tasks are related to instruction behaviors (e.g., Cushion et al., 2012). According to SDT tenets, our findings show, specifically in youth Mexican athletes, that when a coach provides CF to his/her athletes, it can influence their perceptions of vitality as long as the feedback is perceived as legitimate and BPNs are met. This is a significant contribution of this study, so the coaches must first provide CF that is accepted by the athletes (Mouratidis et al., 2010) and provide reasons as to why the quality of the activity or process must be improved, so it is perceived as legitimate. Moreover, coaches must contribute to make athletes feel autonomous (consider the players’ perspective and provide them with various solutions), competent (to have clear and achievable objectives known by athletes), and related (all athletes must be taken into consideration and ought to receive CF). Therefore, in sport, it is also important for coaches to ensure that their players have a high degree of acceptance of the CF they have provided and that their BPNs are met (Vergara-Torres et al., 2020).

Limitations of the study include the fact that participants are from one sport and within a specific age range, and all were boys. Future research should include a larger sample size, a variety of sports, and both sexes. Moreover, since studies on CF by the coach from the SDT perspective are limited, it is important to continue testing the complete sequence of the SDT. Lastly, the mediation model proposed by this study must be tested with samples of athletes from other countries to test the generalizability of the model in other cultures.

It is concluded that CF from the coach and the acceptance of CF by soccer players are contextual variables that predict the satisfaction of BPNs, and this in turn, predicts players’ subjective vitality. Moreover, when CF is perceived as legitimate, it has positive effects in soccer players and improves the quality of their sports participation. Lastly, it is relevant that coaches focus in meeting the psychological needs of the players, due to it positive impact in the psychological well-being, relationships, respect between players and coach, and the sense of belonging to the team.

## Practical Applications

Players perceive their coaches’ corrective behaviors (task and mastery centered) positively (e.g., Álvarez et al., 2019). These corrections ultimately help them to develop their sport more effectively (e.g., Álvarez et al., 2010). What we bring to this research is empirical evidence that it is important that coaches devote effort to relate to their players from an autonomy supportive style, ensuring that they understand the reasons that lead them to the corrections for behavioral changes in their sport. They need to be clear and precise and give players enough time to improve. The players will understand these corrections as legitimate when they feel their needs and emotions are recognized, they can choose between different alternatives of solution to the corrections, they have a fluid communication with their coach, giving them the opportunity to understand the logic and objectives of these corrections. Then, that CF will increase their perception of autonomy, competence, and relatedness with their teammates, which will improve their experience of sports practice through their feeling of vitality. We do recommend seminars, workshops, and training programs for coaches in order to practice and develop these interpersonal skills.

## Data Availability Statement

The datasets presented in this article are not readily available because additional studies are undergoing using the dataset. Requests to access the datasets should be directed to octavio.alvarez@uv.es.

## Ethics Statement

Ethical review and approval was not required for the study on human participants in accordance with the local legislation and institutional requirements. Written informed consent to participate in this study was provided by the participants’ legal guardian/next of kin.

## Author Contributions

JT, RR-E, JL-W, and OA contributed to the conception and design of the study and wrote the first draft of the manuscript. RR-E and JL-W organized the database and performed the statistical analysis. JT, RR-E, JL-W, JZ, MN, and OA wrote sections of the manuscript. MN and OA contributed to global review of the article and relevance of the translation. All authors contributed to manuscript revision, read and approved the submitted version.

### Conflict of Interest

The authors declare that the research was conducted in the absence of any commercial or financial relationships that could be construed as a potential conflict of interest.
